# Air-Assisted Dome Drainage in Acute Corneal Hydrops: A 3D-OCT-Guided Approach

**DOI:** 10.3390/bioengineering12080867

**Published:** 2025-08-12

**Authors:** Antonio Moramarco, Matteo Elifani, Marian Sergiu Zimbru, Andrea Rosolia, Maurizio Mete, Luigi Fontana

**Affiliations:** 1 Ophthalmology Unit, Dipartimento di Scienze Mediche e Chirurgiche, Alma Mater Studiorum University of Bologna, 40126 Bologna, Italy; matteo.elifani@studio.unibo.it (M.E.); marianzimbru@gmail.com (M.S.Z.); maumete@gmail.com (M.M.); luifonta@gmail.com (L.F.); 2 IRCCS Azienda Ospedaliero-Universitaria di Bologna, 40138 Bologna, Italy; 3 Eye Clinic, Multidisciplinary Department of Medical, Surgical and Dental Sciences, University of Campania Luigi Vanvitelli, 80129 Naples, Italy; drrosolia@gmail.com

**Keywords:** ACH, intraoperative OCT, 3D visualization

## Abstract

To describe a technique for managing acute corneal hydrops in eyes with keratoconus using dome stromal drainage with intracameral air injection under real-time three-dimensional (3D) microscope-integrated optical coherence tomography (OCT) guidance. We describe a retrospective case series of six eyes from six patients with keratoconus who developed acute corneal hydrops. All eyes underwent intracameral air injection with controlled dome puncture for stromal fluid drainage, without the use of sutures. The procedure was performed using a 3D visualization system that enables integrated and simultaneous viewing of the surgical field and intraoperative OCT scan (a 3D digitally assisted visualization system that displayed a split-screen view of the surgical field and OCT cross-sections simultaneously). Postoperative resolution of edema and improvement in clarity were documented. The resolution of corneal edema allowed for subsequent mushroom-shaped penetrating keratoplasty performed with a femtosecond laser in four eyes of four patients. All six eyes showed significant resolution of corneal edema within 2 to 4 weeks. Stromal clefts collapsed rapidly after drainage. In each case, the thick edema was reduced to a confined leucoma. No intraoperative or postoperative complications were observed. All four eyes that underwent a femtosecond laser-assisted mushroom-shaped penetrating keratoplasty showed optimal anatomical and functional success. Air-assisted dome drainage, combined with simultaneous 3D and OCT visualization, is a safe and effective technique for treating acute corneal hydrops. This technology enables real-time decision-making and enhances surgical precision, opening the door to advanced procedures that are otherwise limited by corneal opacity.

## 1. Introduction

Acute corneal hydrops (ACH) is a sight-threatening ophthalmic condition that occurs in patients with corneal ectatic disorders. It is characterized by the rapid onset of intrastromal edema due to a rupture in Descemet’s membrane (DM). In these patients, the structural fragility of the cornea, combined with excessive mechanical stress, leads to a full-thickness break in the DM, allowing aqueous humor from the anterior chamber to enter the corneal stroma abruptly. This results in the separation of the stromal lamellae, the formation of intrastromal clefts, and a marked increase in corneal thickness [1,2,3]. The majority of acute corneal hydrops (ACH) cases are associated with keratoconus, the most common form of corneal ectasia. In this context, hydrops is a relatively rare complication, occurring in approximately 2.6–2.8% of patients, or 1.43 per 1000 patients, according to epidemiological studies [1,3,4].

Patients at highest risk of developing ACH are typically those with progressive keratoconus, often younger individuals, who present with atopy and chronic eye rubbing, both of which are thought to increase the risk of DM rupture [5]. Clinically, ACH presents with acute ocular pain, photophobia, conjunctival hyperemia, tearing, and a sudden decrease in visual acuity in the affected eye [6].

On slit-lamp examination, patients with acute corneal hydrops typically present with a focal area of corneal opacification, often exhibiting a bullous appearance. Signs of ocular surface inflammation, such as conjunctival hyperemia and perilimbal injection, may also be present. The fellow eye frequently shows clinical signs of corneal ectasia, including abnormal corneal morphology, Vogt’s striae, or apical scarring [1].

Anterior segment optical coherence tomography (AS-OCT) reveals characteristic findings, including marked stromal thickening and the presence of multiple hyporeflective cavities corresponding to intrastromal clefts. In some cases, a tear in DM can be directly visualized; when sufficiently large, the tear may display rolled or retracted edges. In addition to its diagnostic capabilities, AS-OCT plays a crucial role in monitoring disease progression and guiding clinical decision-making throughout the course of hydrops [7].

In most patients, corneal hydrops undergoes a slow but spontaneous resolution, although the process is often protracted. Resolution primarily depends on two mechanisms: reattachment of the detached DM to the posterior stroma and migration of endothelial cells across the defect to re-establish endothelial continuity [7,8,9].

Historically, treatment for corneal hydrops has been non-surgical. Conservative management includes the use of hypertonic saline to promote stromal dehydration, lubricating drops or ointments to protect the ocular surface, topical antibiotics to prevent secondary infection, and cycloplegic agents to reduce pain from ciliary spasm [10]. Patients are advised to avoid eye rubbing and are closely monitored for early detection of complications, such as infection or perforation. Despite maximal medical therapy, visual outcomes are often suboptimal, and a surgical approach is frequently required to restore visual function [11].

While surgical intervention may not always improve long-term visual outcomes, it has been shown to significantly shorten symptom duration and accelerate recovery, leading to an increasing interest in interventional approaches for acute hydrops [12].

Therefore, over the past two decades, the attention has increasingly shifted toward surgical techniques that aim to accelerate hydrops resolution and preserve vision. One such technique is pneumatic descemetopexy, which involves the injection of an intracameral air or gas bubble, typically a non-expansile mixture of 14% perfluoropropane (C3F8) or 20% sulfur hexafluoride (SF6). The gas serves to tamponade the torn DM, promoting apposition of the edges and preventing further influx of aqueous humor into the stroma. Intracameral gas injection is often combined with stromal venting incisions to facilitate egress of the intrastromal fluid. Despite no statistically significant improvement in visual acuity being reported, this approach can significantly reduce the duration of corneal edema and promote more rapid anatomical and functional recovery [3,12,13].

In selected cases, corneal compressive sutures, placed either full-thickness or intrastromally and oriented perpendicular to the DM tear, can be added to further support reapposition of the membrane, reduce aqueous inflow, and accelerate stromal edema resolution [14,15,16,17]. However, this technique has potential drawbacks, including the risk of perforation, infection, induced irregular astigmatism, neovascularization, and the need for a second procedure to remove the sutures. Therefore, their use is generally reserved for selected cases, depending on the tear morphology and surgeon preference [18].

Despite aggressive management, corneal transplantation remains necessary to restore proper visual function in the majority of patients following acute hydrops, especially when persistent corneal opacity, neovascularization, or irregular astigmatism limits visual rehabilitation [1,3,7].

Currently, deep anterior lamellar keratoplasty (DALK) is preferred over penetrating keratoplasty (PK) in suitable cases, due to a lower risk of rejection and better refractive outcomes. However, DALK requires preservation of both the endothelium and deep stromal layers. In eyes with significant scarring or endothelial compromise, an alternative to conventional PK is mushroom-shaped penetrating keratoplasty (MPK), which involves using two different trephination diameters for the anterior and posterior lamellae. This technique offers the dual benefit of improved refractive results and a reduced risk of immunologic rejection. Historically, MPK was performed by manually dissecting the donor tissue and trephining the graft in two stages, which introduced technical challenges and increased the risk of interface-related complications, such as inner button displacement. Today, femtosecond laser-assisted MPK has become increasingly common, allowing for precise and reproducible dissection of both donor and recipient tissue. Notably, the laser can be used to prepare a mushroom-shaped donor button from a single graft block, avoiding the need for multiple tissue segments and improving surgical reliability [19].

Intraoperative optical coherence tomography (iOCT) has become an increasingly valuable tool in anterior segment surgery [20]. In the context of acute corneal hydrops, it plays a pivotal role in guiding surgical management, allowing for real-time visualization of the corneal stroma, DM, and anterior chamber [21]. This facilitates the optimal placement of the tamponade gas and allows for immediate assessment of its effect on the DM. In addition, iOCT can guide the targeted drainage of intrastromal fluid collections and help assess the impact of compressive sutures [22].

In more complex procedures requiring maximal precision, combining iOCT with three-dimensional (3D) head-up visualization systems offers further advantages, such as the ability to view both the microscopic and tomographic images on the same display, while improving surgical ergonomics [23].

While intraoperative OCT has been increasingly adopted in anterior segment procedures, its integration with digital 3D surgical visualization systems remains poorly explored, particularly in the ACH. To our knowledge, no previous studies have systematically described the intraoperative advantages and workflow improvements offered by this combination.

This synergistic approach enables simultaneous real-time tomographic feedback and stereoscopic depth perception, allowing surgeons to visualize surgical planes with greater precision while maintaining their attention on the operative field. In addition to improving intraoperative comfort and posture, the system may support more controlled maneuvers, particularly during delicate steps such as stromal puncture, dome decompression, and positioning of gas tamponade.

We report a case series in which we applied a sutureless, air-assisted dome drainage technique for ACH under integrated 3D-OCT visualization, demonstrating the feasibility and clinical potential of this combined imaging platform in enhancing intraoperative control and surgical safety.

## 2. Materials and Methods

The study was designed as a retrospective interventional case series. Six eyes from six patients with keratoconus and confirmed ACH were included. All patients were treated at the Cornea Unit of the Ophthalmology Unit, IRCCS Azienda Ospedaliero-Universitaria di Bologna, Bologna, Italy, between December 2023 and December 2024. The surgical procedures were executed using a digitally assisted 3D visualization platform combined with a microscope-integrated OCT (Proveo 8 microscope with an intraoperative OCT EnFocus device (Leica Microsystems, Wetzlar, Germany) (NGENUITY 3D visualization system, Alcon Laboratories, Fort Worth, TX, USA).

This study was conducted in accordance with the Declaration of Helsinki and the CE AVEC: 901/2022/Oss/AOUBo. Informed consent was obtained from all subjects. We included patients who presented with acute corneal hydrops (ACH) in a setting of keratoconus disease, confirmed by means of slit-lamp examination (SLE), corneal topography, and AS-OCT. Exclusion criteria included the following: (1) any history of previous ocular surgery in the affected eye, including prior interventions for hydrops (e.g., sutured drainage, gas tamponade); (2) the presence of significant co-existing ocular conditions such as herpetic keratitis, advanced glaucoma, or severe dry eye disease; and (3) clinical or imaging evidence of ocular infection. A summary of the inclusion and exclusion criteria is presented in Table 1.

The collected data included the following: pre-operative best corrected visual acuity (BCVA) and corneal pachymetry (topographic maps, CASIA 2 (Tomey, Nagoya, Japan). The extent of corneal edema was assessed by evaluating the distribution of intrastromal hyporeflective spaces using OCT imaging (CASIA 2, Tomey, Nagoya, Japan).

All patients were visited at 1 day, 1 week, and 4 weeks after the hydrops surgery.

The primary outcome evaluated was the reattachment of the DM and the resolution of corneal edema, as documented via AS-OCT at the 4-week postoperative follow-up.

The patients underwent MPK using a femtolaser-guided approach (FEMTO LDV Z8 platform; Ziemer Ophthalmic Systems) 3 months after the surgical procedure described.

Given the limited sample size and descriptive nature of the study, statistical analysis of changes in visual acuity was not performed.

### Surgical Technique

**Figure 1:**
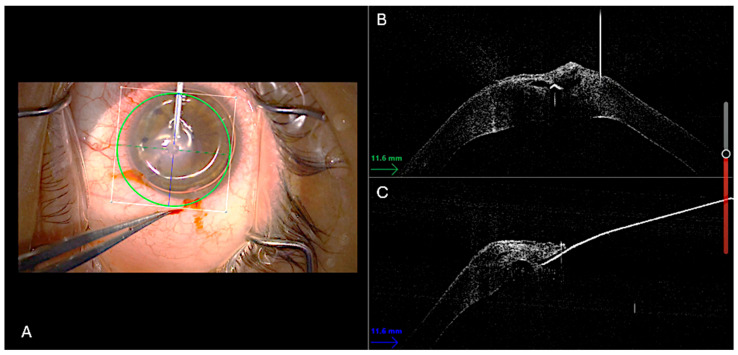
Surgical technique displayed. Intraoperative 3D-OCT split-screen capture showing simultaneous en-face and cross-sectional views. (**A**) An air bubble has already been injected into the anterior chamber, as shown in the en-face image. In the cross-sectional images (**B**,**C**), the 23G sclerotome is visualized as a hyperreflective sign positioned right on the intrastromal cleft.

All procedures were performed in the operating room under sterile conditions by two experienced surgeons (A.M. and L.F.). Under peribulbar anesthesia, two paracenteses and a subsequent inferior iridotomy were performed. The procedure was performed under general anesthesia when necessary (e.g., noncompliant patient). Air was then injected into the anterior chamber, and tamponade of the DM rupture was verified in real time. Using the integrated 3D-iOCT system, precise and controlled drainage of the stromal dome was performed with a 23-gauge sclerotome, allowing for dynamic monitoring of fluid outflow (Figure 1).

No compressive sutures were applied. The paracenteses were sutured with 10-0 nylon. A final intracameral air injection was performed at the end of the procedure. AS-OCT visualization confirmed Descemet’s membrane adhesion to the corneal stroma and complete evacuation of intrastromal fluid.

After the final intracameral air injection, patients were instructed to maintain a supine position for approximately 2 h to optimize the tamponade effect of the air bubble on Descemet’s membrane and promote its reattachment. Postoperative therapy included a topical antibiotic-steroid combination, tapered over 4–6 weeks.

## 3. Results

A total of six eyes of six patients with keratoconus complicated by ACH were included (four men and two women; mean age 22.5 ± 4.59 years). Three eyes were the right eye. Four patients suffered from severe atopic dermatitis that was poorly controlled by medical therapy; two of these four patients had Down syndrome. Two patients had no underlying systemic conditions. The patients’ demographics and baseline data are summarized in Table 2.

The surgical procedure occurred between 5 and 8 days after the outbreak of the corneal hydrops.

The mean pre-operative BCVA was 1.75 ± 0.36 logMar. Postoperatively, at the four-week follow-up visit, all eyes demonstrated a complete resolution of stromal edema, as confirmed by slit-lamp examination and AS-OCT images. A marked thickness reduction of the central cornea was observed. The mean corneal thickness decreased from 848 ± 52.34 μm to 588.17 ± 47.37 μm, as evaluated on the tomographic maps. A concomitant improvement in BCVA was observed, with the mean postoperative BCVA being 1.15 ± 0.31 logMAR. The DM reattached in all cases. No complications were noted, including corneal infection or elevation of intraocular pressure (IOP). The results are summarized in Table 3.

Of the total six patients, four underwent a subsequent femtosecond laser-assisted mushroom penetrating keratoplasty (MPK) under general anesthesia around 3 months after the air-assisted drainage procedure. The mean final BCVA was 0.68 ± 0.15 logMar with a mean astigmatism refractive result of 2.65 ± 0.75 diopters and central corneal thickness (CCT) of 547 ± 20.6 μm. The results are summarized in Table 4.

The two remaining patients did not undergo keratoplasty surgery, in accordance with the patients’ families.

### Case Highlight

**Figure 2:**
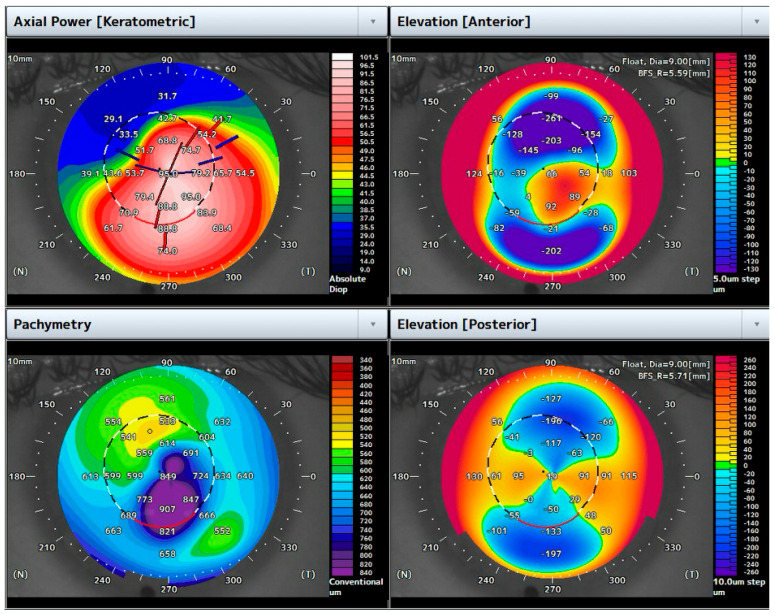
Topographic maps of the case highlighted. Note the increased central corneal thickness due to corneal edema in the setting of ACH.

A 31-year-old male with a previous diagnosis of keratoconus presented with acute dense central hydrops in the left eye. The BCVA was 1.5 logMar, and the corneal pachymetry was 849 μm (Figure 2).

**Figure 3:**
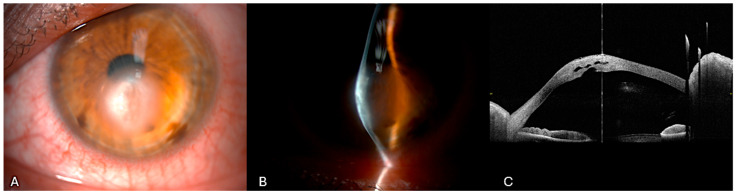
Acute corneal hydrops of the case highlighted. (**A**) Slit-lamp diffuse illumination view of corneal hydrops in advanced keratoconus showing corneal edema at the apex of the cone. (**B**) Narrow slit-lamp view of image (**A**). (**C**) Anterior segment optical coherence tomography showing a pronounced corneal ectasia with increased corneal thickness. The intrastromal hyporeflective spaces are consistent with stromal edema secondary to aqueous influx through the rupture.

He had no history of previous cross-linking or other corneal surgery. Pre-operative AS-OCT highlighted the DM tear with stromal thickening and multiple hyporeflective intrastomal clefts filled with fluid (Figure 3).

**Figure 4:**
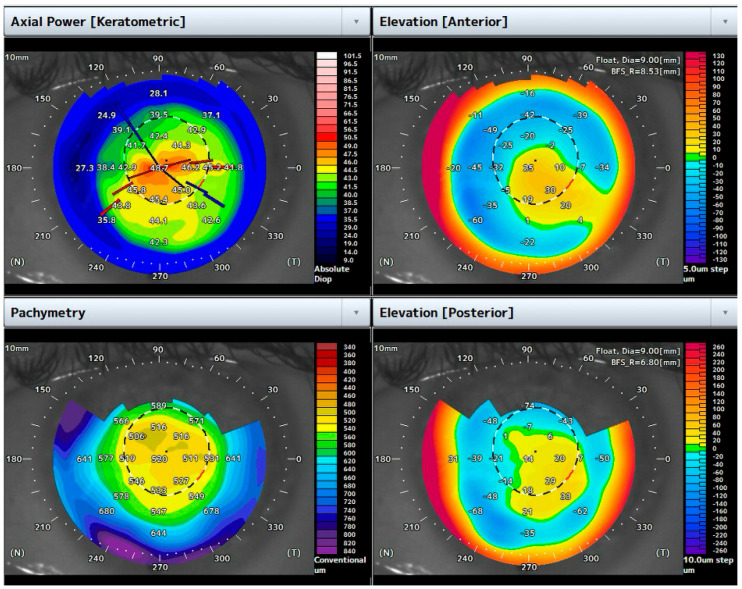
Topographic maps one month after the air-assisted dome puncture. Note the reduction in corneal thickness and the flattening of corneal shape.

He underwent the air-assisted dome drainage procedure, supported by 3D-OCT split-view imaging. Four weeks after the procedure, the BCVA improved to 0.7 logMAR, and the central corneal pachymetry measured 520 μm (Figure 4).

**Figure 5:**
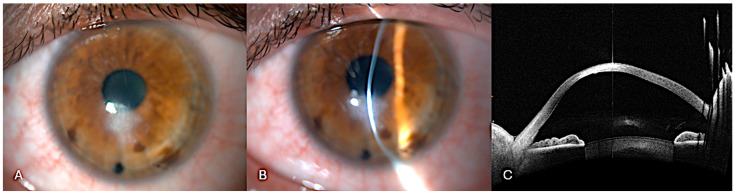
One month after the air-assisted dome puncture. (**A**) Slit-lamp diffuse illumination view of the resolution of corneal edema and scarring. (**B**) Narrow slit-lamp view of image A. (**C**) Anterior segment optical coherence tomography showing how corneal thickness has significantly decreased compared to the pre-treatment scan. Descemet’s membrane appears reapposed in most parts. The overall curvature remains irregular but shows signs of structural recovery. Residual scarring is still evident.

Indeed, the corneal edema regressed to a compact corneal leukoma with reduced dimensions (Figure 5).

**Figure 6:**
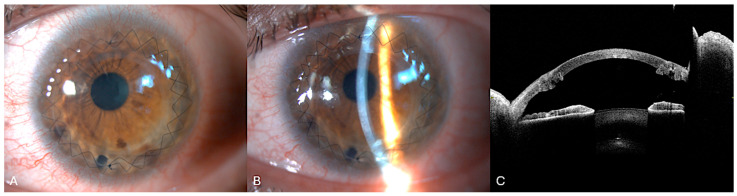
One month post-femtosecond laser-assisted MPK. (**A**) Diffuse illumination slit-lamp showing the graft clarity. Double continuous 10.0 nylon sutures are still present (**B**). Narrow slit-lamp view. (**C**) Anterior segment optical coherence tomography. The graft–host junction is clearly visualized. Note the single-piece lenticule obtained using a femtosecond laser.

No complications were detected during the follow-up visits. Three months after the drainage procedure, a femtosecond laser-assisted MPK was performed using femtosecond laser-assisted trephination and graft preparation, achieving excellent alignment and no interface irregularity. The final BCVA was 0.8 logMAR with an astigmatism of 3D, evaluated 1 month postoperatively. The single-piece corneal lenticule was regular and clear with a perfect match between the donor flap and the corneal host (Figure 6).

This case demonstrates how synchronized live and cross-sectional imaging can convert an otherwise non-operable eye into a candidate for high-precision corneal grafting. The efficacy and safety of the procedure in resolving intrastromal edema and reducing its size subsequently enabled the performance of a femtosecond laser-assisted keratoplasty, ensuring an optimal anatomical and functional outcome for the patient.

## 4. Discussion

The surgical management of ACH is constantly evolving toward more targeted, minimally invasive, and image-guided approaches aimed at reducing edema resolution time and optimizing tissue integrity [24] recovery. In this study, we present a sutureless technique that combines dome-shaped stromal fluid drainage with intracameral air tamponade, performed under simultaneous visualization of the surgical field and tomographic images using a microscope integrated with OCT and a 3D digital viewing system. A key innovation in our approach is the concomitant visualization of both the operative field and the OCT scans. This integration permits true real-time visualization of the intraoperative maneuvers, allowing the surgeon to track the entry of the blade into the stromal clefts, monitor the fluid outflow, and confirm DM reattachment without stopping the surgical procedure.

The use of iOCT in corneal surgery has been extensively described. It has demonstrated marked usefulness, particularly in lamellar keratoplasties. It provides the surgeon with enhanced control over the depth of trephination during a big bubble DALK, while also offering guidance on the accuracy of descemetorhexis, correct graft orientation, and interface quality during endothelial keratoplasty [20].

On the other hand, few reports have described the application of a 3D visualization system in corneal surgery. Cano-Ortiz et al. showed that the NGENUITY visualization system can be successfully used during Descemet’s membrane endothelial keratoplasty (DMEK) surgery [25]. However, we found that the greatest advantage of using a 3D digital viewing system was its integration with real-time iOCT, which provided immediate intraoperative feedback on a single screen during the procedure. The simultaneous use of 3D technology, which enables a heads-up position for the surgeon, and iOCT technology allows the surgeon to perform surgical maneuvers without needing to divert their gaze from the operative field. The exclusive use of intraoperative OCT, although ensuring greater precision in movements within the surgical field, requires the surgeon to look away from the microscope eyepieces. This interrupts the surgical maneuvers and results in a temporally fragmented procedure.

The efficacy of this integrated visualization system is supported by our results: all six patients achieved resolution of corneal edema at the 4-week follow-up control without complications. This procedure ensured rapid anatomical and functional success in all operated cases, enabling the subsequent performance of a penetrating keratoplasty using the mushroom technique with femtosecond laser assistance in four cases. The final anatomical and functional outcome was excellent, with a significant improvement in the patients’ quality of life.

Different surgical techniques have been described as effective for managing acute corneal hydrops, and the recent literature highlights the growing preference toward more targeted and tomographic-guided procedures [24].

iOCT has significantly improved accuracy in combined procedures involving the use of sutures and gas injection, contributing to achieving positive outcomes [22]. In our cases, however, we opted to avoid the use of sutures to preserve the minimally invasive approach of this surgery and to avoid irregular stromal scarring. Despite this, no complications such as intrastromal migration of air occurred.

A similar sutureless approach was previously described by Vajpayee et al., who reported successful management of ACH using pre-operatively obtained OCT images to guide the stromal drainage [26].

Recently, much like in our experience, in a single case report by Kumar et al., iOCT allowed surgeons to perform a sutureless procedure with favorable results [27]. Additionally, our technique benefited from the use of the NGENUITY 3D system, which allowed the surgeon to monitor structural changes in real-time.

Historically, the keratoplasty procedure performed on such eyes has consisted of a penetrating keratoplasty, mainly when carried out shortly after the onset of corneal hydrops. More recently, the use of DALK has also been described and found to be effective in these eyes, but a corneal scar involving Descemet’s membrane usually complicates the procedure [28].

Solving acute corneal hydrops with surgical conservative management means postponing the execution of a keratoplasty in the treated eyes and developing the best surgical strategy [24]. Our sutureless procedure facilitated the execution of an MPK instead of a conventional full-thickness PK in eyes with a posterior corneal scar. Four eyes underwent a femtosecond laser-assisted MPK. Thus, this approach enabled a more tissue-conserving technique, developed to integrate the optical benefits of a large-diameter anterior lamellar transplant with the enhanced graft survival linked to a limited substitution of the corneal endothelium [29]. An additional benefit observed with our technique was the regression of corneal edema into a limited central/paracentral corneal leukoma, which subsequently allowed the trephination of a 6 mm basal button using femtosecond laser technology. The persistence of a larger-diameter leukoma would have made femtosecond laser trephination unfeasible, as described in the literature [30].

Compared to conventional surgical approaches for acute corneal hydrops, such as sutured stromal drainage, gas tamponade alone, or compressive sutures, our technique offers several potential advantages. First, the integration of real-time 3D-OCT visualization allows for precise localization and drainage of stromal clefts, as well as immediate confirmation of Descemet’s membrane reattachment, without interrupting the surgical workflow. Second, the sutureless design reduces the risk of complications associated with stromal sutures, such as neovascularization, induced astigmatism, or infectious keratitis. Finally, the minimally invasive nature of the procedure facilitates faster anatomical recovery and preserves corneal architecture, thereby improving the conditions for subsequent corneal grafting. This more targeted and controlled approach to edema management not only limited the extent of stromal scarring but also enabled the performance of a femtosecond laser-assisted mushroom-shaped penetrating keratoplasty in selected cases. Although a detailed evaluation of graft-related outcomes was beyond the scope of this study, we believe that the ability to perform this type of keratoplasty following edema resolution represents a meaningful added value of our surgical strategy. Nevertheless, long-term follow-up beyond the post-keratoplasty period could be the focus of future longitudinal studies.

We acknowledge several limitations of this study, including its retrospective nature and small cohort size, which reflect the rarity of ACH. Moreover, further studies are needed to confirm the beneficial effects and safety of this procedure.

## 5. Conclusions

In conclusion, the surgical procedure we propose, air-assisted dome drainage under real-time 3D-OCT visualization, resulted in a safe and favorable clinical outcome for acute hydrops in keratoconus. The rationale behind this approach was also to preserve corneal architecture and reduce scarring, thus enabling more precise and advanced keratoplasty techniques, such as femtosecond laser-assisted mushroom-shaped grafts (MPK). Most importantly, this technique highlights how advanced imaging technologies can help the surgeon perform complex maneuvers intraoperatively with real-time feedback on the intraoperative maneuvers.

The retrospective nature and the small sample size of this case series, although reflecting the rarity of acute corneal hydrops, limit the generalizability of our findings. This work should be regarded as a pilot study aimed at demonstrating the feasibility and safety of the proposed approach. Comparative studies with standard treatment approaches are warranted in future investigations.

## Figures and Tables

**Table 1:** Inclusion and exclusion criteria.

Inclusion Criteria	Exclusion Criteria
1. Acute corneal hydrops (ACH) in the context of keratoconus	1. Previous ocular surgery in the affected eye (including procedures for hydrops)
2. Confirmation by means of slit-lamp examination, corneal topography, and AS-OCT	2. Co-existing ocular diseases (e.g., herpetic keratitis, advanced glaucoma, and severe dry eye)
	3. Signs or suspicion of ocular infection

**Table 2:** Demographics and baseline data. M = Male; F = Female; RE = Right eye; LE = Left eye.

Case	Sex	Age	Laterality	Systemic Disease
**1**	M	31	LE	None
**2**	M	16	RE	Down syndrome, Atopic dermatitis
**3**	F	24	RE	Atopic dermatitis
**4**	M	18	LE	Down syndrome
**5**	M	26	LE	None
**6**	F	20	RE	Atopic dermatitis

**Table 3:** Pre-operative and postoperative data of the described surgical procedure. BCVA = Best corrected visual acuity; DM = Descemet’s membrane.

Case	BCVA Pre-Op (logMar)	Pachymetry Pre-Op (μm)	BCVA Post-Op (logMar)	Pachymetry Post-Op (μm)	Pachimetry Improvement (%)	DM Reattachment	Complications
**1**	1.5	849	**0.7**	**520**	**38.7%**	**Yes**	**No**
**2**	2.3	921	**1.6**	**658**	**28.5%**	**Yes**	**No**
**3**	1.6	784	**1.3**	**596**	**24.0%**	**Yes**	**No**
**4**	1.9	853	**1.3**	**617**	**27.7%**	**Yes**	**No**
**5**	1.3	795	**1**	**564**	**29.1%**	**Yes**	**No**
**6**	1.9	886	**1**	**574**	**35.2%**	**Yes**	**No**

**Table 4:** Outcomes post-Femtosecond laser-assisted MPK. BCVA = Best corrected visual acuity; CCT = Central corneal thickness.

Case	BCVA (logMar)	Astigmatism (diopters)	CCT (μm)
**1**	0.80	3.00	550
**3**	0.63	1.80	528
**5**	0.80	2.30	603
**6**	0.50	3.50	507

## Data Availability

The original contributions presented in this study are included in the article. Further inquiries can be directed to the corresponding author.

## Abbreviations

The following abbreviations are used in this manuscript:

ACH	Acute corneal hydrops
DM	Descemet’s membrane
AS-OCT	Anterior segment optical coherence tomography
MPK	Mushroom-shaped penetrating keratoplasty
iOCT	Intraoperative optical coherence tomography
3D	Three-dimensional
DALK	Deep anterior lamellar keratoplasty
PK	Penetrating keratoplasty
DMEK	Descemet’s membrane endothelial keratoplasty
IOP	Intraocular pressure
SF6	Sulfur hexafluoride
C3F8	Octafluoropropane
